# Heterogeneity in dog population characteristics contributes to chronic under-vaccination against rabies in Guatemala

**DOI:** 10.1371/journal.pntd.0010522

**Published:** 2022-07-07

**Authors:** David Moran, Danilo Alvarez, Loren Cadena, Julie Cleaton, Stephanie J. Salyer, Emily G. Pieracci, Leila R. Camposeco, Sulma Bernal, Ryan M. Wallace

**Affiliations:** 1 Unit of Ecology and Epidemiology, Arbovirus and Zoonoses Program, Center for Health Studies, Universidad del Valle de Guatemala, Guatemala City, Guatemala; 2 Centers for Disease Control and Prevention, Central America and Caribbean Region, Guatemala City, Guatemala; 3 Centers for Disease Control and Prevention, National Center for Emerging and Zoonotic Infectious Disease, Poxvirus and Rabies Branch, Atlanta, Georgia, United States of America; 4 Programa Nacional de Zoonosis, Ministerio de Salud Pública y Asistencia Social, Guatemala City, Guatemala; 5 Sistema Integral de Atención en Salud, Ministerio de salud Pública y Asistencia Social, Guatemala City, Guatemala; Universitetet i Oslo, NORWAY

## Abstract

Guatemala has held dog rabies mass vaccination campaigns countrywide since 1984, yet the virus remains endemic. To eliminate dog-mediated human rabies, dog vaccination coverage must reach at least 70%. The Guatemala rabies program uses a 5:1 human:dog ratio (HDR) to estimate the vaccination coverage; however, this method may not accurately reflect the heterogeneity of dog ownership practices in Guatemalan communities. We conducted 16 field-based dog population estimates in urban, semi-urban and rural areas of Guatemala to determine HDR and evaluate the standard 5:1. Our study-derived HDR estimates varied from 1.7–11.4:1 (average 4.0:1), being higher in densely populated sites and lowest in rural communities. The community-to-community heterogeneity observed in dog populations could explain the persistence of rabies in certain communities. To date, this is the most extensive dog-population evaluation conducted in Guatemala, and can be used to inform future rabies vaccination campaigns needed to meet the global 2030 rabies elimination targets.

## Author summary

Standard dog population estimates used for planning and evaluating dog vaccination campaigns in Guatemala are prone to error due to spatial and ownership heterogeneity among dog populations; advanced population estimation methods should be implemented in areas with persistent endemicity of dog-mediated rabies.

## Introduction

Rabies is a viral disease that has the highest case-fatality rate (99.9%) of any known infectious disease [1]. Every year more than 59,000 people die from rabies [2,3], with the majority (99%) of these cases being dog-mediated. Rabies can be controlled and eliminated in dog populations by obtaining ≥ 70% vaccination coverage rate in susceptible populations [4]. Many countries worldwide have achieved this vaccination goal and realized elimination of the canine rabies virus variant [5]. However, there are still an estimated 120 countries where dog-mediated rabies is endemic, including Guatemala where approximately three dog-mediated human rabies deaths are detected by health authorities annually [6]. In order to plan effective and efficient mass vaccination campaigns, the size and ownership characteristics of the dog population must be known [7]. This information helps determine the total number of vaccine doses needed, as well as the overall campaign budget, staff, and implementation strategy [8]. While international guidance recommends that rabies control programs estimate dog populations to plan effective targets for vaccination and meet the global elimination targets for canine-mediated rabies [9], many countries have not conducted canine population estimates due to the cost, limited resources, lack of expertise, and loosely defined methodologies [8].

There are several methods described to estimate dog populations [10–15] including household surveys, dog counting, and census amongst others; however, the output of most methods need to be compared to the representative human population for broad extrapolation to regional or national rabies control programs [11]. Rabies programs typically describe dog populations in terms of the human to dog ratio (HDR), and oftentimes these ratios are extrapolated beyond the community in which they were derived with little thought as to the representativeness of the original study population [16]. The HDRs are even sometimes used to generalize dog populations between countries, when in-country estimates are not available [16]. However, studies show the HDR is not constant across all communities and can differ greatly based on variables that include community characteristics, religion and poverty [17–19].

Even in Latin America, ratios differ within and between countries. For example, an HDR in Mexico was reported as 4.6 [20,21], and Bolivia reported an HDR of 4.6 [22]. Brazil (4.0), Argentina (5), Ecuador (7.6), Haiti (9), and Chile (6.6) all have varying estimates of their dog population, making it challenging to rely on regional rather than national and sub-national level data for vaccination campaign planning [23–26].

Since 2010, the Guatemalan Ministry of Health (MoH) has conducted annual vaccination campaigns using a HDR of 5:1 to estimate the dog population when procuring vaccines, planning campaign logistics, and estimating post-vaccination levels of herd immunity [27]; this estimation assumes the HDR is homogenous across all communities. This information has not been validated in the field, but was adopted from data derived from neighboring countries [28]. Studies of canine populations conducted in different regions within Guatemala give evidence that this ratio is variable [29–31]. However, these studies were relatively small in scale, developed for wildlife ecological studies, and utilized differing methodologies (e.g single day dog counting or household surveys), which makes comparison and interpretation of outcomes for the national vaccination program challenging.

The variability in canine population estimates could partially explain why cases of rabies persist in certain communities of Guatemala, despite vaccination coverages reported to be at or above 70%—the level that needs to be achieved to eliminate dog-mediated rabies [9]. The main objective of this study was to explore the spatial and ownership heterogeneity of dogs in different scenarios in Guatemala to provide a better approach for the evaluation of the variation of HDRs in the country. For this, we compared field and household methods for dog population estimation that have been previously used in canine rabies endemic settings, including some regions of Guatemala [11,12,31]. These methods include sight-resight surveys (SRS) [15], household surveys (HHS) and a combination of both methodologies in eight health areas of the country, encompassing 16 communities, including urban, semi-urban and rural settlements. The results of this study highlight the heterogeneity in dog populations and dog ownership characteristics across Guatemala, which resulted in under-vaccination of dogs in rabies endemic communities.

## Methods

### Ethics statement

All the procedures and protocols were classified as non-research by the Institutional Ethics Committee, and the Animal Use Care Committee from the University del Valle (UVG) (protocol No. 147-08-2016), and verbal consent was obtained from the participants.

### Study area and community selection

Guatemala’s health system is divided into 29 health area. Each area, integrated by the set of health establishments and services, constitutes a management level that leads, coordinates and articulates all health services in their jurisdictional area. National health policies and national health planning are develop and applied using the divisions that has its own programing, monitoring and evaluation activities [32]. We reviewed official Ministry of Health surveillance reports and selected communities with a high dog bite incidence compared to the national average (greater than 80 persons bitten per 100,000 inhabitants) [32]. Four of the eight health areas were additionally considered high-risk for rabies based on reports of (i) at least one rabid dog reported in the last three years, or (ii) at least one dog-mediated human rabies case reported in the last 15 years. From these, we randomly selected 16 communities, located in eight health areas, to ensure that we had representative communities from places with a history of rabies cases (Table 1). The selected communities were categorized as urban (n = 5), semi-urban (n = 3), and rural (n = 8) according to criteria described by the UN for Latin American countries [33], and a site code was assigned to each community (Table 1). Further considerations for site selection included accessibility and safety according to local authorities.

**Table 1:** Characteristics of the selected sites.

Health Area	Community	Site code	Community type	Evaluation Method	Human population*	Dog bite incidence** (annual/100,000 habitants)	Dog rabies reported in the past 3 years**	Human rabies cases in the past 15 years**
Huehuetenango					1,381,970	172	Yes	6
	Petatan	Urban 1	Urban	SRS	10,850			
	Jacaltenango	Urban 2	Urban	SRS	8,000			
Sololá					533,345	115	Yes	3
	Panajachel	Urban 3	urban	SRS	11,142			
Suchitepéquez					595,986	81	Yes	3
	Churirin	Rural 1	Rural	Combined	623			
	Rosario Patulul	Semi-Urban 1	semi urban	Combined	900			
Guatemala sur					1,076,598	121	Yes	1
	Villa Nueva Centro	Urban 4	urban	Combined	3,224			
Santa Rosa					406,925	95	No	0
	Hawaii	Rural 2	Rural	HHS	679			
	El Rosario	Rural 3	Rural	HHS	283			
	Las Quechas	Rural 7	Rural	Combined	270			
	El Pumpo	Rural 8	Rural	Combined	1,373			
	Monterrico	Semi-Urban 3	semi urban	Combined	6,594			
Peten Sur Occidente					279,723	138	No	0
	La Romana	Rural 4	Rural	HHS	651			
	Sabaneta	Rural 5	Rural	HHS	969			
	San Marcos	Rural 6	Rural	HHS	626			
Sacatepéquez					365,474	241	No	0
	Santa Catarina Barahona	Semi-Urban 2	semi urban	SRS	3,654			
Zacapa					244,881	116	No	0
	Gualan	Urban 5	urban	SRS	8,200			

* Instituto Nacional de Estadística, Guatemala 2018.

** Programa Nacional de Zoonosis, Ministerio de Salud Pública y Asistencia Social, Guatemala 2019.

The method of dog population enumeration was selected based on availability of staff and resources at each community, which prohibited the equal distribution of enumeration methods along rural, semi-urban, and urban communities.

### Sight-resight surveys (SRS)

We conducted sight-resight (SRS) surveys according to previously published methods [12]. The UVG staff, MoH staff, and veterinary students from the National University were trained on the SRS method by U.S. Centers for Disease Control and Prevention (U.S. CDC) staff. Enumeration teams consisted of two persons, one person to record pictures and data from the dogs sighted, and one person to record the evaluation area of the transect annotating in the paper forms. Within the selected communities a transect was defined in which field surveyors walked through the community for 2–4 hours depending on the transect size recording characteristics of all dogs sighted. This process is repeated on the following day with a focused effort to identify dogs seen on the previous days. The enumeration staff were equipped with a Garmin Montana 680t GPS unit, and a paper data recording form. Dog enumerators captured an image of each dog sighted using the GPS unit’s camera and collected information on the location, physical description (sex, age, color, visible marks, any physical characteristics that helps to identify the animal), presence of a collar, and health status of each dog sighted (evident signs of disease or illness or not). Additionally, dogs were categorized as ‘newly sighted’ or ‘previously sighted’ based upon the memory recall of the survey team, and for the analysis the dog photographs were used to confirm the field observations. Dogs were recognized by every single animal unique marks, including animal color, missing ears, scars, limping etc, and/or identifier such as collars [15].

For the SRS surveys, the dogs were counted and classified as described in previous studies using the Lincoln-Petersen equation with Chapman’s bias correction [12,15]. To estimate the total number of free-roaming dogs within each community (beyond the transect that was surveyed), counts were adjusted using the sampling fraction of the human population in the transect (the proportion of the total reported population in the area that was inhabiting the selected households), extrapolated to the total human population in the community [34] Human population along the transect was determined by using GIS and satellite to define the inhabited area in the community surveyed and human population assessed. [35].

### Household surveys (HHS)

We conducted household surveys selecting the households starting in the first street of the communities, and using a skip pattern of two households [12]. We interview the first adult living in the house who consent to participate, and verbal consent was obtained before starting the survey. The information was collected on paper forms filled out by experienced and trained staff, as described previously [15]. The approximately 10 minute interviews were conducted in Spanish, asking for number of dogs owned, number of persons living in the household, and number of dogs allowed to freely roam. Where HHS and SRS methods were jointly conducted, selected households were located along the same transects as where the dog counts were conducted. If a selected household owner declined to participate or was not home at the time of the visit, the nearest household was visited instead.

The data from the HHS were used to determine the number of dogs owned, the number of persons per household, and the number of owned dogs allowed to freely roam in the study areas. With this, the dogs were classified as owned always confined (OCD) (dogs that was not allowed to freely roam and live in a household that ensures they do not have contact with stray dogs); owned semiconfined (OSD) (dogs that were allowed to roam in some periods of time without their owner supervision); and owned never confined dogs (OND) (dogs that were allowed to roam freely without supervision and/or that lives in a household with free access to the street). Respondents were also asked if anyone in the household provided food to community dogs (CD). To estimate the total number of owned dogs within each community, the number of dogs per household was extrapolated using the sampling fraction of households in the community that were surveyed.

### Combined population analysis

In sites where both SRS and HHS were conducted (gold standard method), data were combined in the following novel stepwise approach to determine the overall dog population demographics:

1. Total, Owned Confined Dogs (OCD_T_) = OCD_S_ / P_HHS_ * P_T_
  OCD_S_ = Owned, Confined dogs claimed in survey P_HHS_ = Human population represented by households surveyed P_T_ = Total human population in the represented community
2. Total, Owned Semi-Confined Dogs (OSD_T_) = OSD_S_ / P_HHS_ * P_T_
  OSD_S_ = Semi-confined dogs claimed as owned by a survey household.
3. Total, Owned Never-Confined Dogs (OND_T_) = OND_S_ / P_HHS_ * P_T_
  OND_S_ = Never-confined dogs claimed as owned by a survey household.
4. Total, Free Roaming Dogs (FRD_T_) = [(C_1_ + 1) + (C_2_ + 1) / (R_e_ + 1)– 1] / P_FS_ * P_T_
  C_1_ = count of dogs on the first day C_2_ = count of dogs on the second day R_e_ = count of dogs sighted on both days, re-sights P_FS_ = Human population represented by the transect surveyed.
5. Total, Community Dogs (CD_T_) = max {FRD_T_−(OND_T_ + OSD_T_), 0}
6. Total Dog Population = OCD_T_ + OSD_T_ + OND_T_ + CD_T_

To estimate the OCD and total dog population in sites where only the SRS method was conducted, the average fractional proportion of the OCD_T_ was calculated within each community-type that conducted the gold-standard [12,16] combined enumeration approach (rural, semi-urban, and urban) and the fractional proportion was applied to the FRD_T_ by the equation:

$$Adjusted\;Total\;Dog\;Population=FRD_{T}/(1‐(OCD_{T}/(OCD_{T}+OSD_{T}+OND_{T}))$$

To obtain the CD and total dog population in sites where only the HHS method was conducted, the average fractional proportion of the CD_T_ was calculated within each community-type that conducted the gold-standard combined enumeration approach (rural, semi-urban, and urban) and the fractional proportion was applied to the total owned dog population by the equation:

$$Adjusted\;Total\;Dog\;Population=(OCD_{T}+OSD_{T}+OND_{T})/(1‐(CD_{T\;(all\;combined\;sites)}))$$

Confidence intervals were calculated for each dog ownership and roaming status, using the "normal approximation" method [36]. HDRs were calculated for FRD_T_ and the Total Dog Population. The fractional proportion of the overall dog population was calculated for OCD_T_, OSD_T_, OND_T_, and CD_T_.

We obtained the human population for each site from the official census [35]. Human population density, the area of the community addressed in the surveys (Km^2^), the Km^2^ of inhabited community for each site were calculated. This data was analyzed by regression to find the association between the total HDR by population density, proportion of roaming dog population by population density, free roaming dog HDR by population density, total dog population density by human population density, and free roaming dog population density by human population density. We considered any empirical dog population estimates with a 95% CI that does not include the official estimate (point estimate of 5 people per dog) to be a significant deviation.

Associations between human and dog populations were tested by logarithmic, linear, an exponential models and the best fit was based on the highest R2 value.

## Results

**Fig 1:**
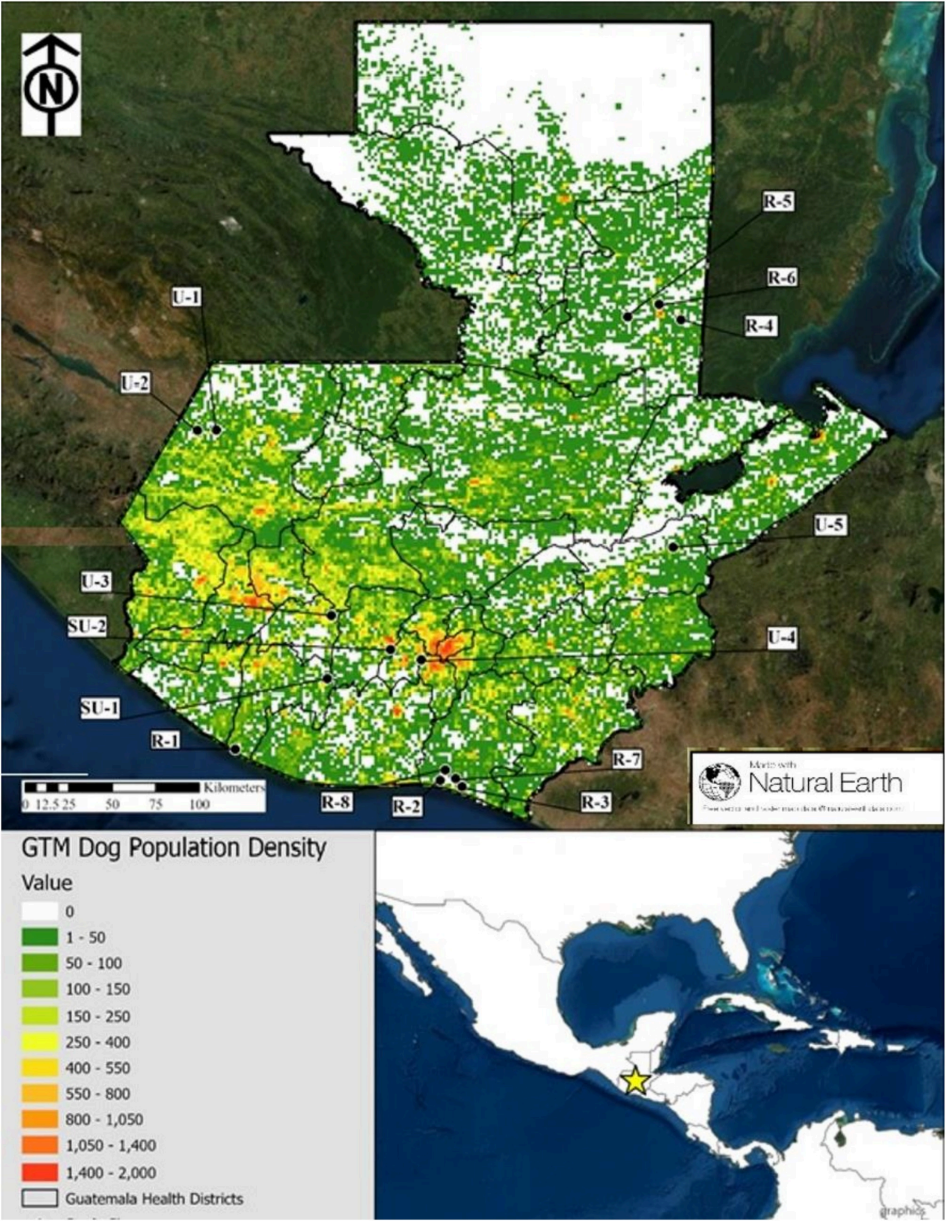
Dog population density as determined by a 16-site dog enumeration study with stratified extrapolation based on human population density (https://www.naturalearthdata.com/tag/imagery/; https://www.naturalearthdata.com/about/terms-of-use/).

**Fig 2:**
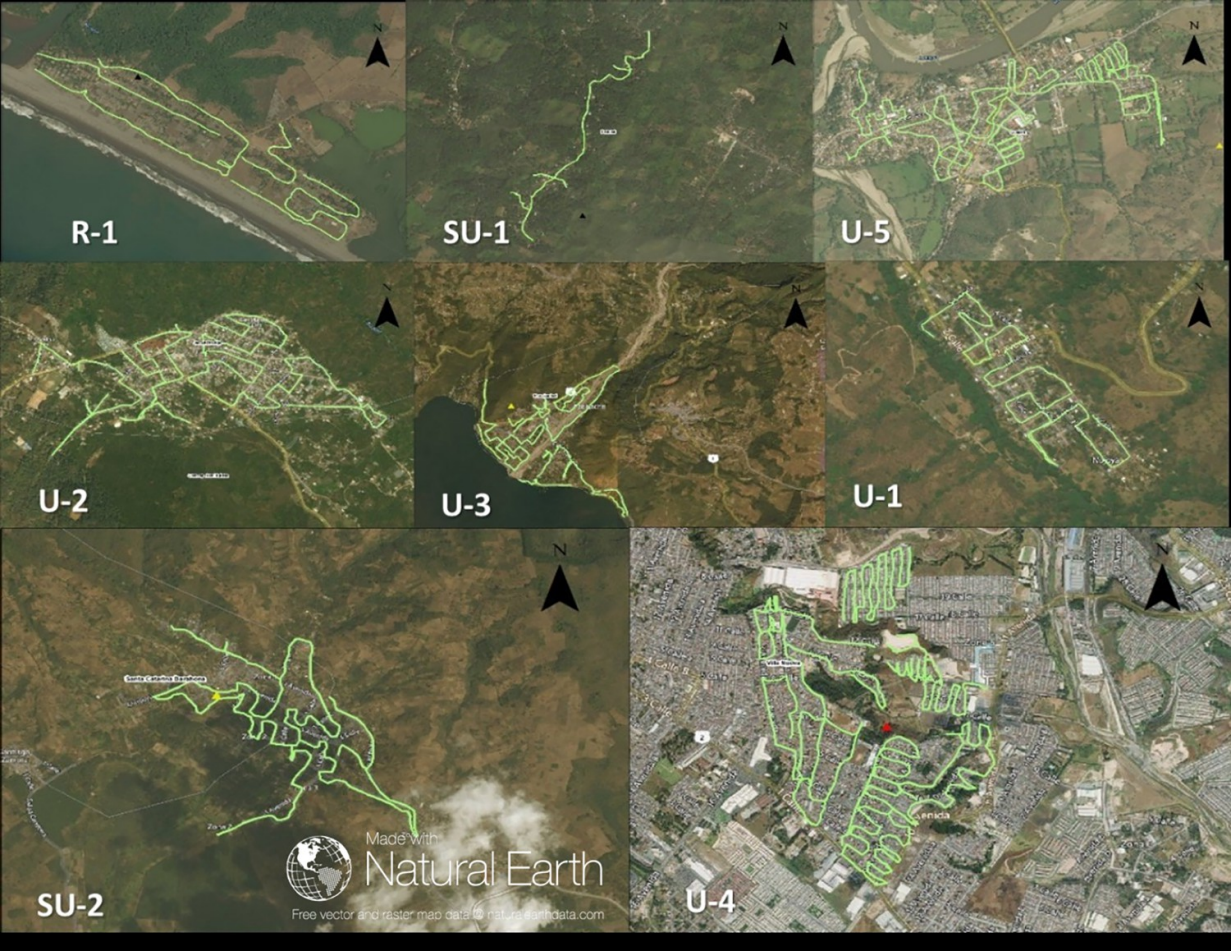
Free roaming counting transects path for SRS method in the study sites. (Base map and data from OpenStreetMap and OpenStreetMap Foundation, and contains information from OpenStreetMap and OpenStreetMap Foundation, which is made available under the Open Database License; https://www.openstreetmap.org/; https://www.openstreetmap.org/copyright). Yellow lines indicates the transect path in the SRS surveys at the study sites. Sites code: R-1 = Chuiririn, SU-1 = El Rosario Patulul, U-5 = Gualan, U-2 = Jacaltenango, U-3 = Panajachel, U-1 = Petatan, SU-2 = Barahona, U-4 = VillaNueva.

We conducted 919 HHS and walked 119 kilometers for SRS between March and July 2018 for a total of 16 community dog surveys: 5 HHS, 5 SRS, and 6 combined HHS and SRS (Tables 2 and 3). The total human population represented in these health areas was 4,884,902 (31% of the total human population of Guatemala). The communities were classified and stratified by urban, semi-urban, or rural; and by evaluation method (Fig 1). The path of the transects in the 8 communities (SRS alone and SRS + HHS) are shown in Fig 2.

**Table 2:** Sight-re sight and household survey sites. Guatemala, March–July 2018.

Site	Interviews*	Persons per house†	Population represented‡	Total human population§	OCD_S_ (%) ‖	OSDs + ONDs (%) ¶	Estimated dog population (95% CI)	Health Area population#	Proportion coverd¬	Dog count 1 **	Dog count 2 ***	Re-sighted dogs ****	Free roaming dog (FRD) estimate (95% CI)	Free-roaming dogs estimate for the total site area (95% CI)
Rural 1	65	5	347	623	12 (14)	75 (86)	156 (128–185)	42,291	2	68	57	41	94 (85–104)	123 (110–135)
Rural 7	29	4.1	93	270	10 (29)	24 (71)	99 (72–125)	29,846	4	61	57	47	74 (70–78)	129 (122–137)
Rural 8	34	4.6	339	1373	53 (38)	87 (62)	567 (495–639)		8	63	71	33	135 (112–157)	181 (151–211)
Semi-urban 3	27	4.7	675	6594	75 (52)	70 (48)	1416 (1212–1621)		16	157	84	53	248 (215–280	975 (848–1102)
Semi-Urban 1	114	6.1	667	900	116 (35)	218 (65)	451 (417–485)	40,683	2	270	190	155	331 (316–345)	358 (343–374)
Urban 4	170	5	878	3224	60 (24)	189 (76)	914 (818–1010)	618,397	4	55	61	33	101 (87–115)	758 (651–866)

* Number of households where one interview was conducted

† Mean of persons living in the surveyed households

‡ Total of persons reported living in the surveyed households

§ Official human population in the surveyed community

‖ Owned confined dogs

¶Sum of owned semi confined dogs and owned not confined dogs

# Official human population in the municipality

¬ Percentage of the area of the community covered by the counting teams in the linear Km transects

** number of dogs sighted on day 1 of survey

*** number of dogs sighted on day 2 of survey

**** number of dogs sighted on day 2 of survey that was previously sighted on day 1.

**Table 3:** Sight–Re Sight survey sites. Guatemala March–July 2018.

Site	Health Area population#	Proportion of the site covered by the survey (linear km)¬	Dog count 1 **	Dog count 2 ***	Re-sighted dogs ****	Free roaming dog (FRD) estimate (95% CI)	Free-roaming dogs estimate for the total site area (95% CI)	Human: FRD-ratio (95% CI)
Semi-Urban 2	3,256	15	56	57	12	253 (150–357)	827 (490–1,165)	4.4 (3.1–7.5)
Urban 1	13,831	4	91	96	43	202 (170–234)	2178 (1,836–2,520)	5 (4.3–6.0)
Urban 2	37,131	20	356	316	133	844 (758–929)	2320 (2,084–2,556)	3.4 (3.1–3.8)
Urban 3	14,022	14	453	413	402	465 (463–468)	759 (755–763)	14.7 (14.6–14.8)
Urban 5	45,663	20	84	97	29	277 (211–342)	625 (477–773)	13.1 (10.6–17.2)

# Official human population in the municipality

¬ Percentage of the area of the community covered by the counting teams in the transects

** number of dogs sighted on day 1 of survey

*** number of dogs sighted on day 2 of survey

**** number of dogs sighted on day 2 of survey that was previously sighted on day 1.

### Human dog ratio evaluation

#### HHS only sites

Five communities, representing a total resident population of 3,208 people, were surveyed by HHS only, with 480 interviews conducted. The average number of people per household was 5.5 (range 3.8–7.4). In total, the survey included information for 2,152 persons in survey households, which claimed ownership of 623 dogs. The unadjusted HDR across these five sites was 3.5 (range 2.1–5.6) (Table 2). The unadjusted HDR level of confidence (95%) included the national level of 5:1 for two of the five communities (40%). Most (93%) dogs in these communities were owned semi-confined (OSD_S_) or owned never confined (OND_S_) (range 76% - 95%) (Table 2). The number of unowned, community dogs could not be assessed with the HHS-only method.

#### SRS only sites

Five communities, representing a total resident population of 41,846 people, including one semi-urban and four urban sites, were surveyed by SRS method only. A total of 73 km of transects were walked while counting dogs in the communities. Within these transects, the total human population was determined to be 15,496 (37% of total resident population), and the free roaming dog population (FRD) was estimated to be 2,041 animals. The unadjusted FRD-HDR for the study sites was 7.6 (range 3.4–14.7) (Table 3). The HDR level of confidence (95%) included the national level of 5:1 for three of the five communities (60%). The rate of dog ownership could not be assessed with the SRS-only method.

#### Combined method sites

Six communities representing a total resident population of 12,984 people were surveyed by both HHS and SRS methods, including three rural, two semi-urban, and one urban. A total of 439 HHS were conducted, representing a total household population of 2,999 persons, with an average of 4.9 persons per house (range 4.1–6.1). The owned dog population was reported by survey respondents to be 989, giving an owned dog HDR of 3.0 (range 2.0–4.7) (Table 2). The SRS survey was conducted along 46 km of linear transect of dog counting, with an estimated FRD population of 982 and human population of 4,591. The FRD-HDR in these communities was 4.7 (range 2.1–7.6) (Table 2).

### Comparison of HDR in rabies present and rabies absent communities amongst rural, semi-urban, and urban scenarios

**Fig 3:**
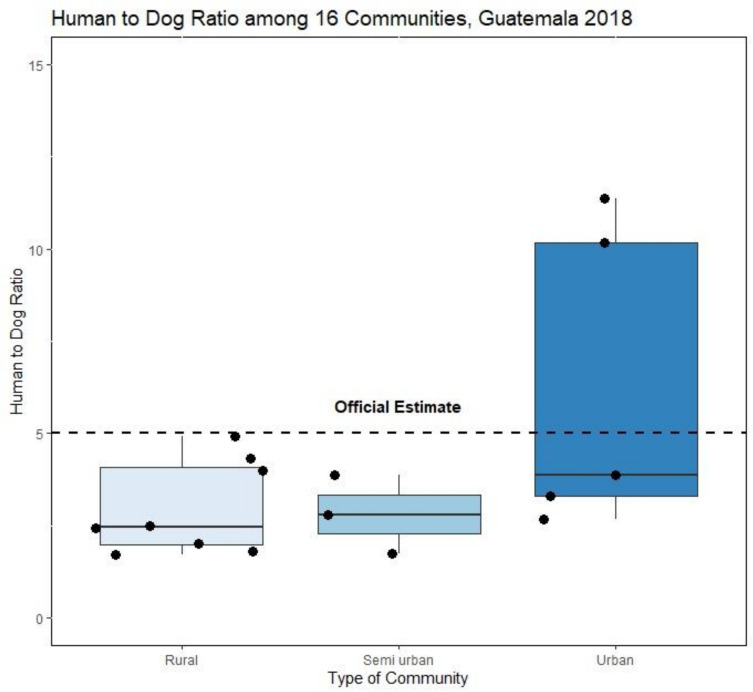
Distribution of the Human:Dog ratio in the different types of surveilled communities in this study, and their comparison with the official 5:1 Guatemala Human:Dog Ratio.

After adjusting the dog populations to account for confined and free roaming dogs in communities that only conducted one survey method, the adjusted HDR (aHDR) in the 16 participating communities varied from 11.4:1 to 1.7:1, being lower in the rural and semi-urban communities than the urban communities (Table 4 and Fig 3)

Rural communities had an aHDR of 3.0 (95% CI 2.4–3.9) and a range of 1.7 to 4.9. Semi-urban communities had an aHDR of 2.8 (95% CI 2.4–4.0), and a range of 1.7 to 3.9. Urban communities had the highest aHDR, with a value of 6.3 (95% CI 5.6–7.3) and a range of 2.7 to 11.4 (Table 4 and Fig 3).

**Table 4:** Differences between the official, unadjusted and adjusted human to dog ratio amongst the study sites–Guatemala, March—July 2018.

Enumeration site	Official Estimate (5:1)	Adjusted Empirical Estimate (95% CI)	Difference (%)	Unadjusted HDR	Adjusted HDR (95% CI)	Rabies Status
R-1	125	156 (120–195)	25%	4.0	4 (5.2–3.2)	Rabies
R-8	275	567 (289–684)	106%	2.4	2.4 (4.8–2)	No Rabies
R-6	125	127 (96–158)	2%	5.6	4.9 (6.5–4)	No Rabies
R-3	57	140 (80–200)	148%	2.3	2.0 (3.5–1.4)	No Rabies
R-4	130	150 (118–183)	15%	5.0	4.3 (5.5–3.6)	No Rabies
R-2	136	375 (305–445)	176%	2.1	1.8 (2.2–1.5)	No Rabies
R-7	54	158 (134–183)	193%	1.7	1.7 (2.0–1.5)	No Rabies
R-5	194	389 (345–434)	101%	2.8	2.5 (2.8–2.2)	No Rabies
SU-1	180	515 (473–556)	186%	1.7	1.7 (1.9–1.6)	Rabies
SU-3	1319	1708 (1425–1991)	30%	3.9	3.9 (4.6–3.3)	No Rabies
SU-2	731	1306 (773–1839)	79%	4.4	2.8 (4.7–2.0)	No Rabies
U-2	1600	2994 (2689–3298)	87%	3.4	2.7 (3.0–2.4)	Rabies
U-1	2170	2811 (2369–3252)	30%	5.0	3.9 (4.6–3.3)	Rabies
U-5	1640	806 (615–998)	-51%	13.1	10.2 (13.3–8.2)	No Rabies
U-3	2228	980 (974–985)	-56%	14.7	11.4 (11.4–11.3)	Rabies
U-4	645	979 (818–1140)	52%	3.3	3.3 (3.9–2.8)	Rabies

*Data from HHS or SRS surveys

† Variation from the official estimation compared with empirical estimation

‡ Sites where HHS AND SRS surveys are considered the Gold Standard

no adjustment was not used in them

§ Estimation based on only one of the methods were adjusted

‖‖ Site classification according to the rabies official reports.

**Fig 4:**
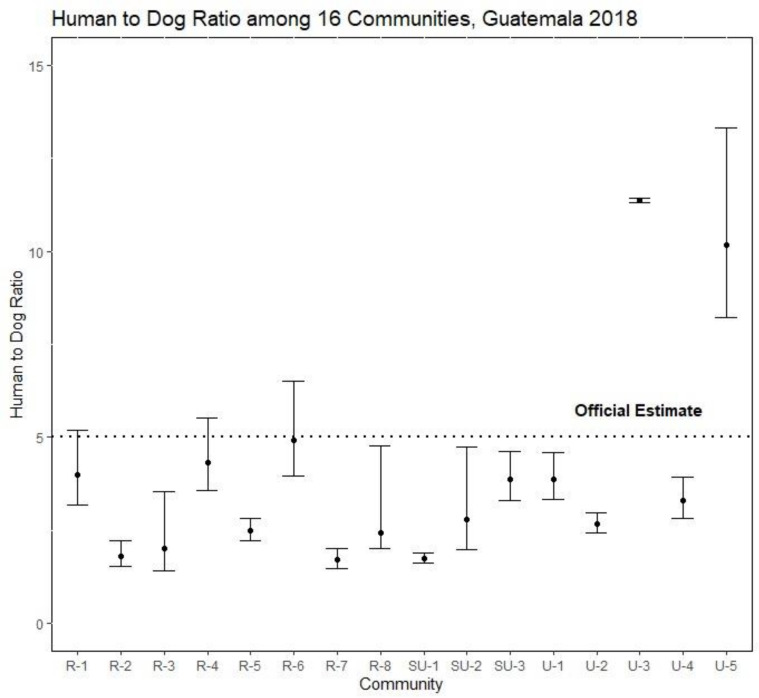
Comparison of the Human:Dog ratio between the different surveilled communities in this study.

There was a large degree of heterogeneity in the final empirical dog population estimations. The empirical population estimation method resulted in significantly lower aHDRs (and therefore higher estimated dog populations) than the national estimate in 10 of the 16 communities assessed (Table 4 and Fig 4). Overall, these 10 communities had dog populations that were 1.6-fold higher than official estimates. This would equate to a 60% decrease in the estimated vaccination coverage; for example, a reported coverage of 80% based on the 5:1 official HDR would be a true coverage of 48% in most rural and peri-urban communities. Two communities had a significantly higher HDR (and therefore lower estimated dog population) than the national estimate (Table 4 and Fig 4).

The rate of under-estimation of the dog population for sites with no documented rabies cases was 124%, compared to the 121% rate of under-estimation in sites with documented rabies cases (Table 1). The rate of under-estimation of the dog population in rural communities was 167%, compared to 179% in semi-urban communities, and 79% in urban communities (Table 4 and Fig 4).

### Comparison of adjusted dog populations between urban, semi-urban, and rural sites

#### Urban communities

**Fig 5:**
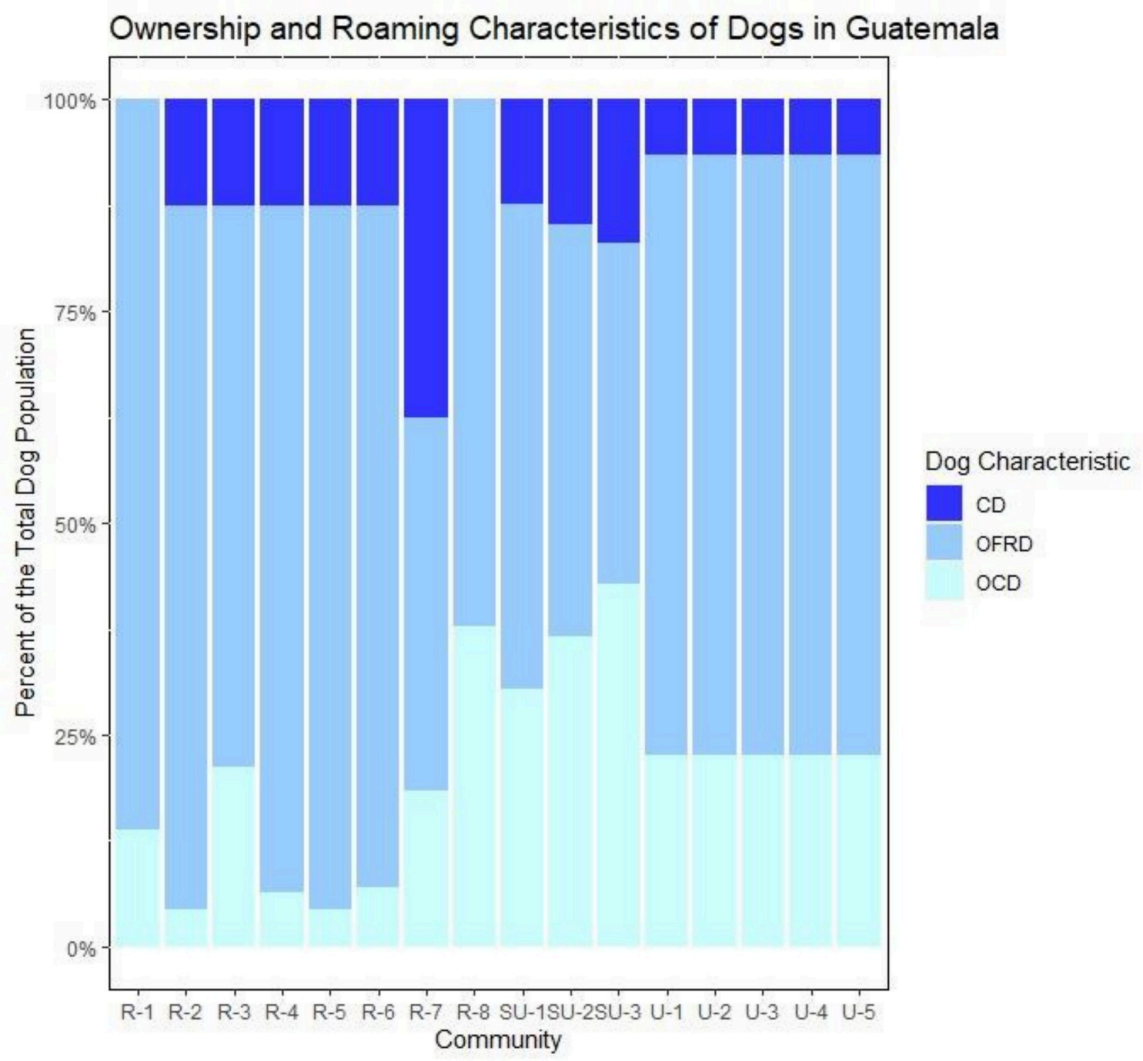
Ownership and roaming characteristics of the dog population among the surveilled communities.

At the urban sites, 23% of dogs were owned confined dogs (OCDs), 71% were owned free roaming dogs (OFRDs), and 7% of dogs were unowned community dogs (CDs) (Fig 5).

#### Semi-urban communities

At the semi-urban sites, 37% of the dogs were OCDs, 48% were OFRDs, and 15% were CDs (Fig 5).

#### Rural communities

In the rural sites, 14% of the dogs were OCDs, 73% OFRDs, 13% were CDs (Fig 5).

### Associations with Human Population Density

**Fig 6:**
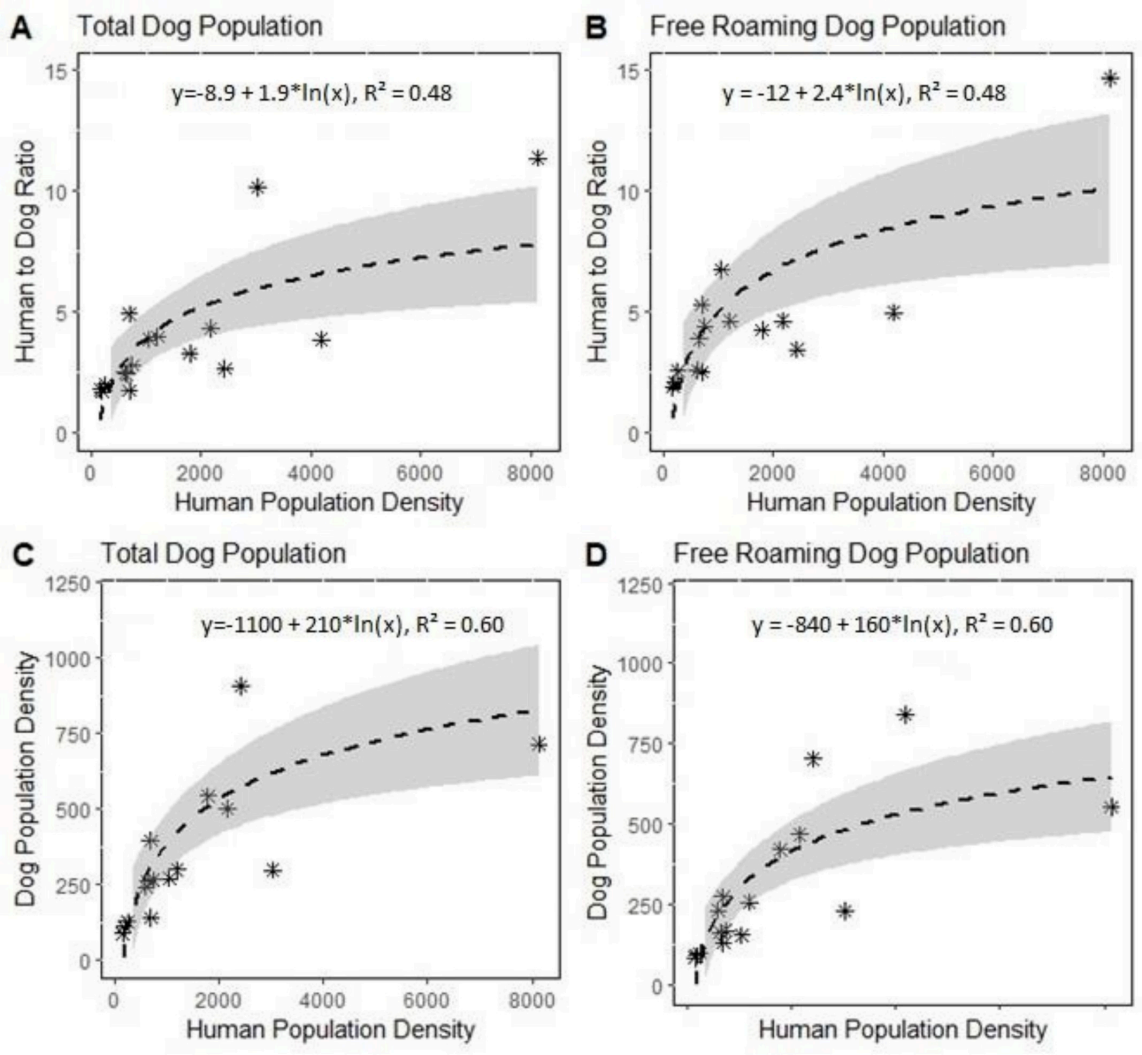
Association between the free roaming dog population density and the human population density amongst the study sites, March—July 2018.

**Fig 7:**
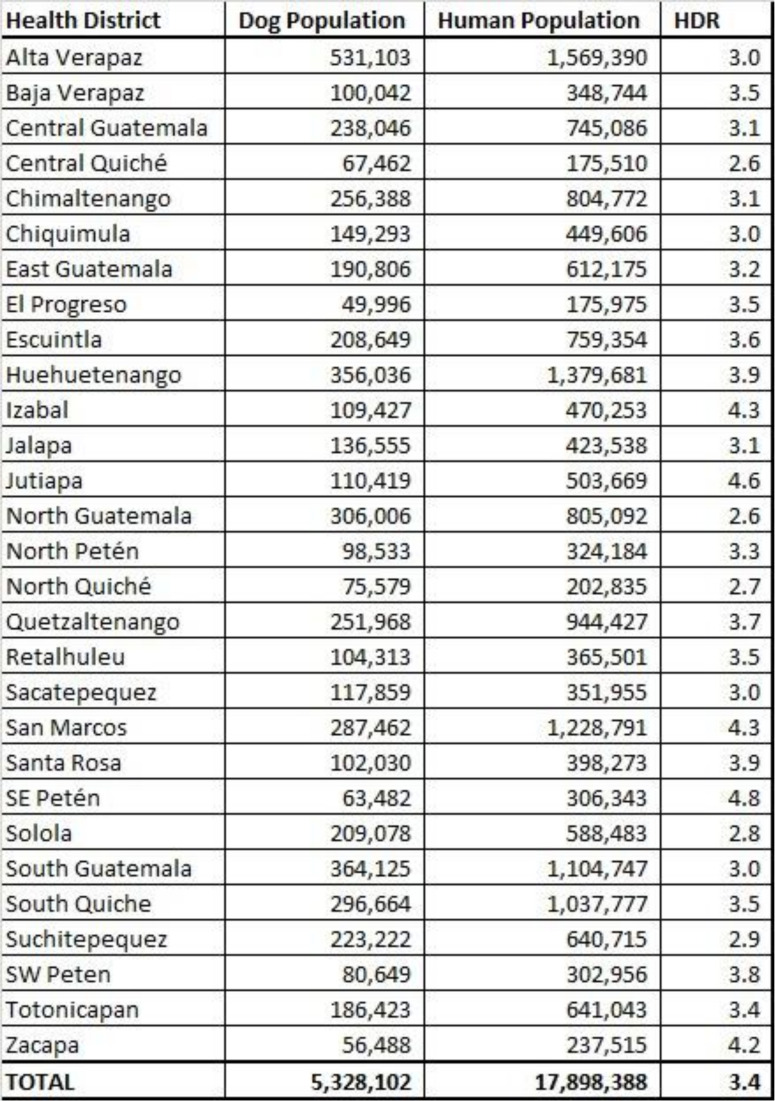
Projected HDR by Guatemala Health Areas based on the study results.

The community’s human population density was positively associated with the aHDR and best fit a logarithmic function (y = -8.9 + 1.9*ln(x), R^2^ = 0.48) (Fig 6A). The adjusted FRD-HDR has strong positive logarithmic correlation with the human population density (y = -12 + 2.4*ln(x), R^2^ = 0.48) (Fig 6B). Dog population density was highly correlated with human population density, showing a positive logarithmic correlation (y = -1100 + 210*ln(x), R^2^ = 0.60) Fig 6C). Free roaming dog population density and the human population density was similarly associated (y = -840 + 160*ln(x), R^2^ = 0.60) (Fig 6D). For the total country extrapolation we selected the functional equation from the dog density by human population density (y = -1100 + 210*ln(x), R^2^ = 0.60), applied to the known Guatemala human population density. The result indicates the aHDR is below the 5:1 used by the Country (Fig 7).

## Discussion

Until now, this is the most extensive evaluation of dog populations in Guatemala, and it goes beyond prior studies that only focused on rural areas and single-method enumeration approaches. The results of the present study support the hypothesis that there is a large degree of heterogeneity in dog populations between communities in Guatemala, demonstrating a high variability of the HDR and dog density among different community types and geographical areas of the country.

Previous studies and surveys in certain rural regions of Guatemala have reported that nearly every household owns at least one dog, and in some cases up to 10 [37], suggesting the 5:1 HDR may result in an underestimation of dog populations amongst semi-urban and rural communities in the country [29,30]. Since post-vaccination herd immunity in Guatemala is evaluated based on a standard HDR estimate, and not through field-based post-vaccination surveys, such underestimations could lead to chronic under vaccination of the dog population. Results from these 16 sites suggest that in certain communities, primarily those in rural settings, vaccination coverages may be nearly two-times lower than what has been reported using the standard 5:1 HDR. Furthermore, in two urban areas the aHDR was higher than the official estimate, meaning the dog populations are overestimated in those places. Given the high variability of dog populations between communities, and the wide range of variation associated with an urban-rural divide, community-specific dog censuses or tailored vaccination methods may be necessary in certain communities with persistent dog-mediated rabies virus infections [7].

Household surveys account for only owned dogs, whereas SRS surveys identify free roaming dogs but cannot distinguish ownership. Either method performed independently risks under-estimating the dog population when there are significant numbers of community dogs (HHS underestimates the population) or owned-confined dogs (SRS underestimates the population). Only when there is good evidence to indicate that either one of these populations is absent from a community, would a single method be appropriate.

All sites had significant populations of owned-confined dogs, meaning that HHS methods were necessary for accurate dog counts in all communities evaluated in this study. Community dogs could only be directly measured in the six sites that performed both enumeration methods; community dogs were identified in four (66%) of these communities. This suggests that SRS methods are likely necessary in most communities in Guatemala if the objective is to fully characterize the dog population. However, the proportion of community dogs is likely variable and the combined methodology approach to enumeration requires additional logistical and financial support. These methods are not foolproof, and biased estimates can arise from non-systematic survey methods such as recall bias, dog miss-identification, and weather conditions such as rain. Therefore, a consistent approach to dog enumeration should be considered, if estimates will be used to inform vaccination strategies throughout Guatemala. In communities with persistent rabies or where community members feed and provide care to community dogs, combined methods may be necessary to accurately enumerate the dog population and plan vaccination activities.

Nearly all study sites we evaluated had a canine population with some degree of under estimation by the national 5:1 ratio. Among the three community-types, only the urban communities had an overall HDR that was statistically indistinguishable from the national estimate. A trend was observed showing that the density of dogs increased as human population density increased (Fig 1). The strong correlation between dog and human densities may offer a more accurate means of estimating dog populations, rather than a standard ratio applied to all community-types and highlights that this correlation is neither fixed nor linear. If resources are not immediately available for field population studies, this relationship can be used to make tailored community estimates of dog populations that can inform both the overall vaccination targets as well as the methods of vaccination applied in the community. It is important to note that there is acluster of data points in lower density communities, as shown in Fig 6. This indicates that our estimates may not be generalizable to the high-density areas, where further dog population assessments may be needed.

Observations in communities from Africa, Indonesia and India, have shown that the dog population size is regulated by human demand [14,38]. In those studies, factors as the density of houses, bakeries and garbage piles were significant predictors of dog population size. This can be easily extrapolated to the rural Guatemalan population, where sanitation services are not as available and ample food sources can be found in streets and open-pit garbage dump sites [34].

In conclusion, estimating dog populations and routinely evaluating vaccination campaigns are important activities for a successful rabies elimination strategy. Advanced population estimation methods should be implemented in areas with persistent endemicity of dog-mediated rabies. The results of this study highlight the heterogeneity in dog populations and dog ownership characteristics across Guatemala, which has likely resulted in under-vaccination of dogs in many rabies endemic communities. Due to the differences that exist in the dog populations in Guatemala, it is necessary to improve local tailored estimates and vaccination coverages, and this is relevant for the regional strategy for rabies elimination in the Americas since Guatemala an Mexico shares 963 km of border [39], and Mexico was declare free of canine rabies in 2019 [40]. Interestingly, this study found that dog populations were more strongly correlated with human population density than the total human population in a community; extrapolations based on this density-dependent relationship may offer a more accurate method for apply study data to obtain national and sub-national dog population estimates. This study suggests that focusing more intensive dog enumeration efforts in communities with persistent rabies cases may be necessary to achieve canine rabies elimination in Latin America.
